# Effectiveness of Total Contact Casting in the Treatment of Neuropathic Plantar Ulcers: An Observational Study

**DOI:** 10.7759/cureus.109479

**Published:** 2026-05-23

**Authors:** Mohit Kataruka, Puja Sannyashi, Souvik Bhattacharjee, Amala Sajeev

**Affiliations:** 1 Physical Medicine and Rehabilitation, All India Institute of Medical Sciences (AIIMS), Kalyani, IND

**Keywords:** diabetes type 2, leprosy, neuropathic plantar ulcers, off-loading, total contact casting

## Abstract

Introduction: Neuropathic ulcers occur mainly at the plantar aspect and correspond to areas with peak plantar pressure, usually seen in a sensory-deficient foot. Trophic ulcers are seen in diabetic neuropathy, post-leprosy, and any other neurological disorders that lead to peripheral sensory loss and ultimately lead to amputation. The treatment principle involves avoiding pressure on the ulcerated site, called 'offloading.' Conventional total contact casting (TCC) is an essential offloading modality for noninfected, non-ischemic neuropathic plantar ulcers. There is a paucity of literature showing the role of TCC in neuropathic foot ulcers. The present study aimed to evaluate the effectiveness of TCC in the treatment of neuropathic plantar ulcers.

Materials and methods: After obtaining ethical clearance, the study was conducted at the Physical Medicine and Rehabilitation Department of a tertiary care center in West Bengal, India, from March 2024 to August 2024. Total contact casting was performed on 16 patients with neuropathic plantar ulcers. Assessment was performed at the first visit (zero weeks) and at the second, fourth, and sixth weeks.

Results: Two-way repeated measures ANOVA was performed. The test revealed a statistically significant difference in ulcer volume between pre- and post-treatment (F = 7.742, p = 0.01). Bonferroni’s test for multiple comparisons found a statistically significant difference in the volumes of ulcers at the first and second follow-up from before the start of treatment. Upon applying split-plot repeated-measures ANOVA, no significant difference was found between the decrease in ulcer volume and the individual’s sex (F=.099, p=0.757).

Conclusions: Total contact casting is an effective, non-invasive method for off-loading a plantar ulcer, though it is time-consuming and associated with minimal complications.

## Introduction

Trophic plantar ulcers are typically seen on the feet with sensory deficits. They most commonly occur in patients with peripheral neuropathy, developing over the plantar surface at areas of maximum pressure. Because of sensory loss, these ulcers are usually painless. Repeated trauma and continuous pressure lead to skin breakdown and subsequent ulcer formation. Weakness of the intrinsic foot muscles and subluxation of the metatarsal heads increase shear stress, leading to callus formation and an increased risk of ulceration [1]. Trophic ulcers are commonly associated with diabetic neuropathy, healed leprosy, and other neurological disorders that cause peripheral sensory loss, which can ultimately lead to amputation if not properly managed [2-5].

The main principle of treatment is avoiding pressure on the ulcerated site, a process known as 'offloading.' Offloading can be achieved through various methods, including complete bed rest, different crutches, a walker, a cane, healing sandals, or total contact casts, with or without a walking iron. Dr. Paul Brand first popularized the use of total contact casting (TCC) in patients with neuropathic Hansen’s disease in Carville, Louisiana, in the 1960s [6]. The cast is usually prepared using a plaster of Paris cast and molded to maintain contact with the sole and the lower leg [7]. The cast supports the foot and lower leg and redistributes pressure over the entire plantar surface (sole) to reduce pressure over the ulcer area [8-10].

In TCC, plaster is applied over the limb to the patellar tendon. This rigid cast distributes weight along the plantar aspect of the foot to the entire lower extremity, thereby relieving pressure on the forefoot and toes during walking and standing, enabling ulcers to heal while still allowing the patient to remain mobile, thus achieving offloading [11]. A removable orthosis can achieve offloading, but achieving strict compliance is challenging.

Several randomized controlled trials have demonstrated that TCC offers either equivalent or superior efficacy compared to other wound care or offloading methods. Consequently, TCC has traditionally been regarded as the gold standard for healing plantar ulcers. However, only about 1.7% of clinical centers utilize TCC, citing challenges such as the time-intensive application process, patient discomfort, and lack of clinician familiarity.

The present study aims to investigate the contemporary effectiveness of TCC in the healing of plantar ulcers. Furthermore, given the limited data on TCC’s efficacy for plantar ulcers, this study also sought to evaluate its effectiveness in these specific ulcer locations. The aims and objectives were to evaluate the effectiveness of TCC in reducing ulcer area and volume among patients with Wagner grade I or II neuropathic plantar ulcers. The secondary objective was to explore whether ulcer-healing response differed by ulcer site and patient sex.

## Materials and methods

This observational study was conducted from March 2024 to August 2024 at the Physical Medicine and Rehabilitation department of a tertiary care center in West Bengal, India. The All India Institute of Medical Sciences (AIIMS; Kalyani, WB, IND) provided institutional ethical clearance (approval no. IEC/AIIMS/Kalyani/certificate/2024/088). Informed and written consent was taken from the patients. We recruited all the patients who reported to the Physical Medicine and Rehabilitation OPD with a plantar ulcer.

Patients were excluded if they had non-plantar ulcers (e.g., ulcers on the dorsum of the foot), ischemic limbs, infected ulcers, osteomyelitis, severe foot deformities due to Charcot arthropathy, or any diagnosed mental disorders. Nineteen patients met the inclusion criteria, of which one was excluded due to the exclusion criteria, and two were lost to follow-up. Thus, a total of 16 patients aged 40 to 60 years with neuropathic plantar ulcers, classified as Wagner grade I or II, were enrolled in this study. Of these 16 patients, 12 had forefoot ulcers, and four had hindfoot ulcers.

**Figure 1:**
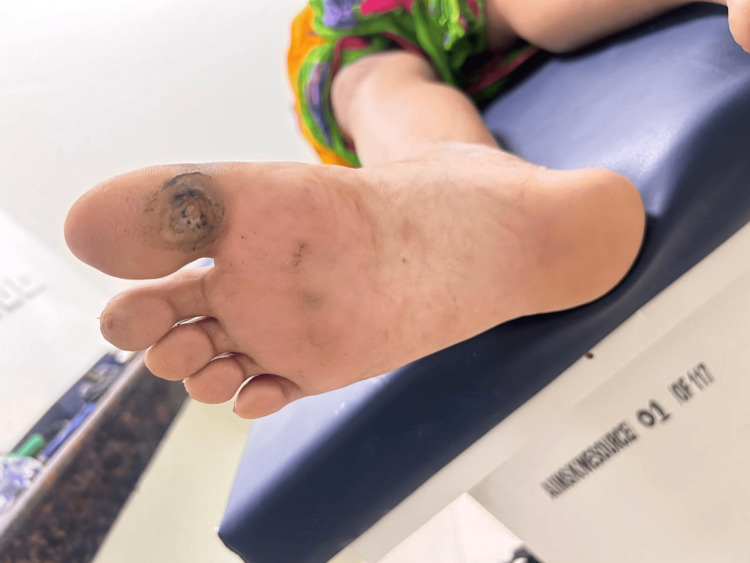
A patient with a forefoot ulcer at the OPD

**Figure 2:**
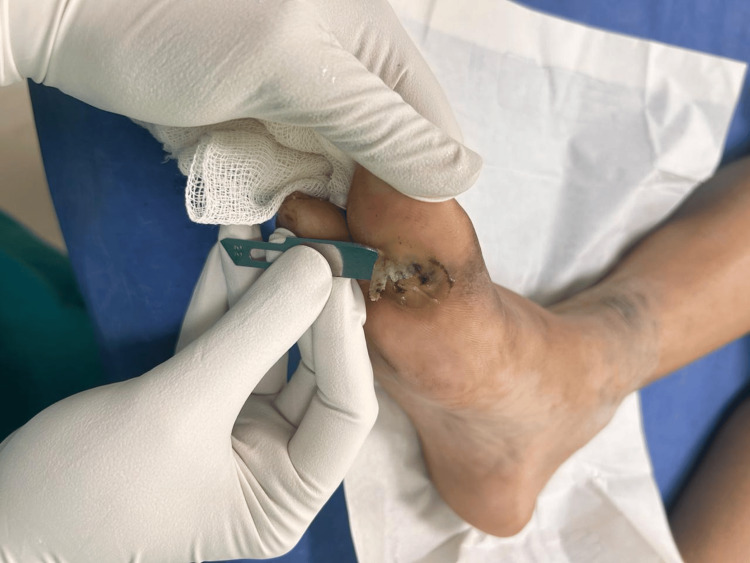
Debridement of an ulcer A patient attending the OPD with a plantar (forefoot) ulcer underwent debridement of the ulcer with surgical blade no.22, which is the first step prior to TCC. TCC: Total contact casting

**Figure 3:**
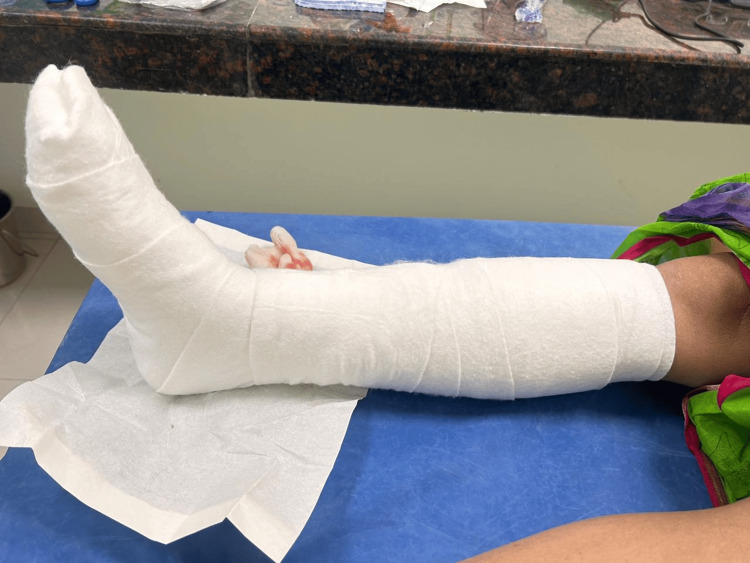
A soft cotton roll wrapped on the leg before casting After debridement of the ulcer (plantar aspect), a soft cotton roll is wrapped around the leg before the application of plaster of Paris for TCC. TCC: Total contact casting

**Figure 4:**
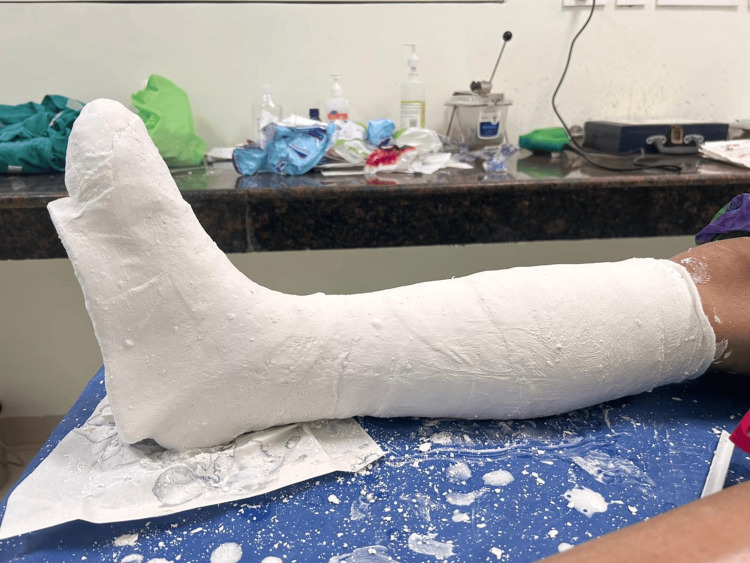
The cast is applied with a wedge at the plantar aspect The plaster of Paris cast was applied over the affected leg up to the patellar tendon with a wedge applied at the plantar aspect in the TCC procedure. TCC: Total contact casting

Offloading interventions were made using TCC to relieve mechanical stress in a specific region of the foot. Each patient who attended the OPD was carefully observed (Figure 1) and underwent surgical debridement of the ulcer (Figure 2). Then, a soft cotton roll was applied over the affected lower limb up to the patellar tendon (Figure 3), followed by plaster of Paris casting of the entire foot and a portion of the lower leg (Figure 4).

**Figure 5:**
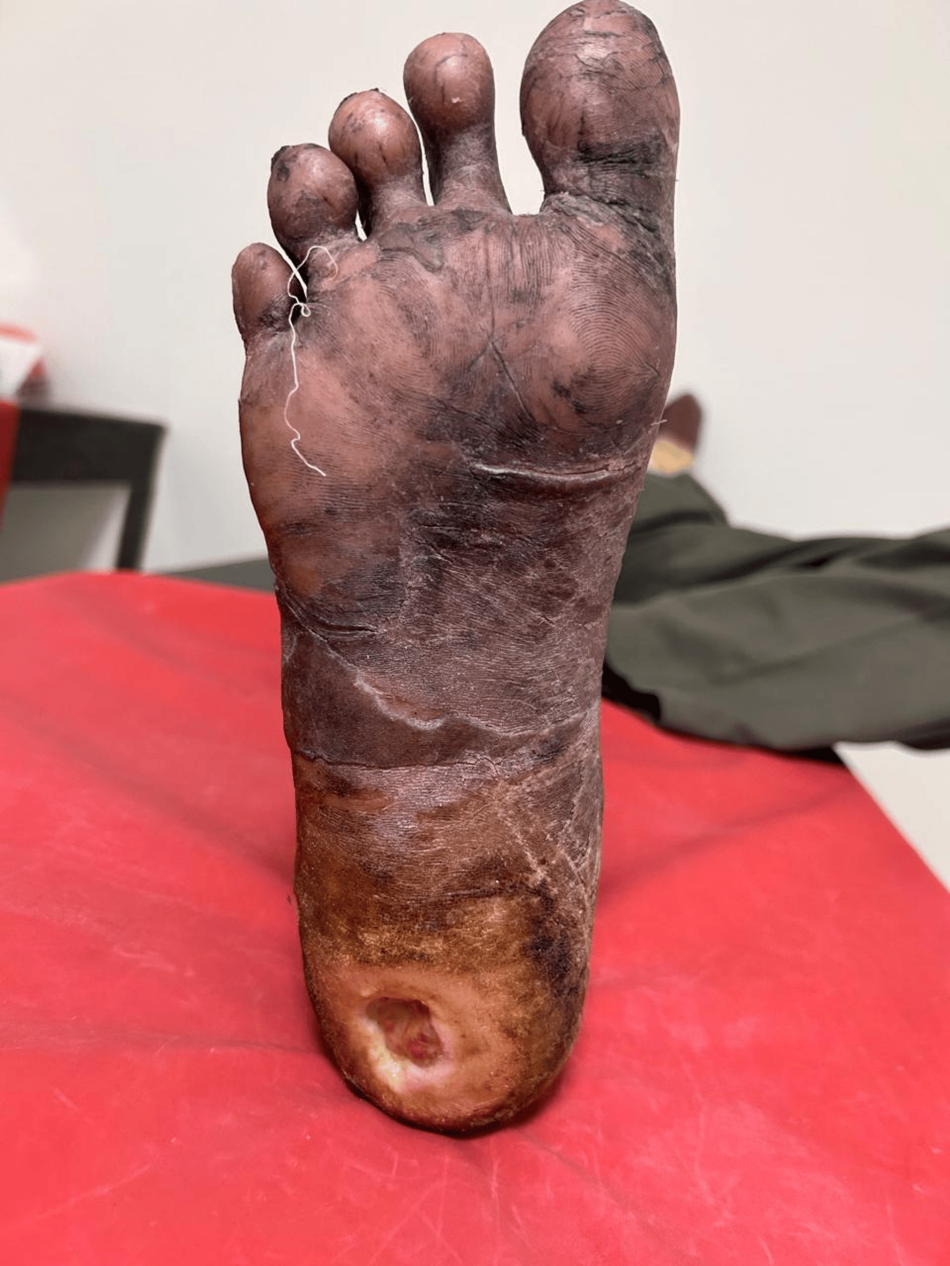
A patient's foot with ulcer reduced in size on the second visit after TCC application The ulcer (plantar hind-foot) was reduced in size from 4 cm x 3 cm (on the first visit) to 2 cm x  1.5 cm on the second visit after TCC application. TCC: Total contact casting

**Figure 6:**
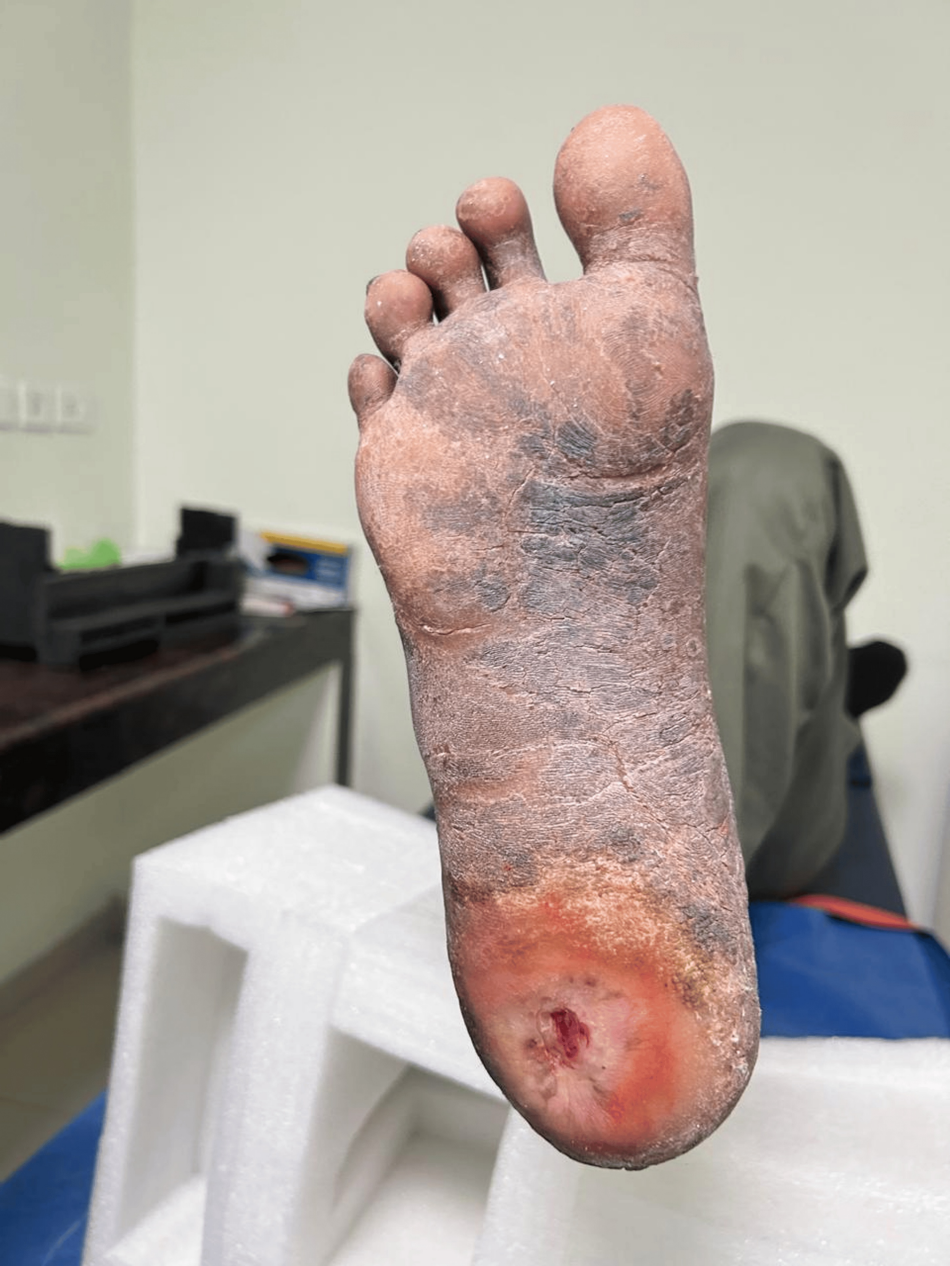
A patient's foot with ulcer reduced in size on the third visit after TCC application The ulcer (plantar hind-foot) was reduced in size from 4 cm x 3 cm (on the first visit) to 1 cm x  0.5 cm on the third visit. TCC: Total contact casting

The total-contact cast was meticulously molded over the entire surface. Casts were removed after 10 days, at which point repeat debridement was performed, and TCC was reapplied. This cycle was repeated four to five times, contingent on the rate of ulcer healing. At each visit, the ulcer dimensions were carefully measured with a scale and recorded (Figures 5-6). Subsequently, the ulcers completely healed. 

Statistical analysis was done using SPSS Statistics version 27 (IBM Corp., Armonk, NY, USA). Categorical variables were presented as percentages, and quantitative data were presented as means and standard deviations. Bonferroni’s test was used for multiple comparisons of volume difference in consecutive visits. The changes in area and volume across subsequent visits were determined using a two-way ANOVA. A p-value of <0.05 is considered statistically significant.

## Results

A total number of 16 patients with diabetic neuropathic ulcers were included in the study, with a male:female ratio of 1:1, a mean age of 53±13 years, and a mean duration of symptoms of 48±15 months. In the majority of the cases (75%), the forefoot was involved (Table 1).

**Table 1:** Baseline characteristics of the study population The continuous variables are presented as mean ± standard deviation, and the categorical variables are presented as proportions.

Parameters	Mean ± SD / ratio
Age	53 ±14
Duration of ulcer (months)	48 ±15
Length (cm)	3.25 ±2.9
Breadth (cm)	2.32 ±1.73
Area (square cms)	11.81 ±19.74
Volume (cc)	10.87 ±19
Gender (male:female)	1:1
Site (forefoot:hindfoot)	3:1
Grade of ulcer (grade I:grade II)	1:3

A two-way repeated-measures ANOVA was performed to determine whether the mean ulcer volume in patients differed between treatment initiation and the subsequent three follow-ups. The test revealed a statistically significant difference in ulcer size before and after treatment (F = 7.742, p = 0.01).

There is a statistically significant reduction in ulcer area between the area at baseline vs. the area at the first follow-up (p = 0.014) and the area at baseline vs. the area at the second follow-up (p = 0.001). The reduction from baseline to the sixth week was also statistically significant (p = 0.001) (Table 2). Comparisons among subsequent visits following the initial volume reduction did not reach statistical significance, except for the visit after two weeks of TCC vs. the visit after six weeks of TCC, suggesting that the maximum therapeutic effect was achieved early after TCC application.

**Table 2:** Data showing significant improvement in the area of reduction of the ulcer A p-value of <0.05 is considered statistically significant; *The marked data showed a statistically significant difference V0: Initiation of treatment, V1: First follow-up after two weeks of TCC, V2: Second follow-up after four weeks of TCC, V3: Third follow-up after six weeks of TCC, TCC: Total contact casting

Period of assessment	Data in mean ± SD	Follow-up visit comparison	p-value (two-way ANOVA)
Initiation of treatment (V0)	11.81±19.67	V0-V1	0.014*
First follow-up after two weeks of TCC (V1)	8.65±17.45	V1-V2	0.055
Second follow-up after four weeks of TCC (V2)	6.59±15.99	V2-V3	0.100
Third follow-up after six weeks of TCC (V3)	5.58±14.65	V3-V0	0.001*
		V0-V2	0.001*
		V1-V3	0.001*

Bonferroni’s test for multiple comparisons revealed a statistically significant reduction in ulcer volume at the first and second follow-ups compared to baseline for plantar ulcers. There is a statistically significant reduction in ulcer volume between volume at baseline vs. volume at the first follow-up (p = 0.014) and volume at baseline vs. volume at the second follow-up (p = 0.001). The reduction from baseline to the sixth week was also statistically significant (p = 0.001) (Table 3). The TCC was effective in significantly reducing ulcer size, particularly during the first two follow-ups. After that period, healing continued, but the rate of improvement was no longer statistically significant.

**Table 3:** Data showing a significant improvement in reduction of the volume of the ulcer A p-value of <0.05 is considered statistically significant; *The marked data showed a statistically significant difference V0: Initiation of treatment, V1: First follow-up after two weeks of TCC, V2: Second follow-up after four weeks of TCC, V3: Third follow-up after six weeks of TCC, TCC: Total contact casting

Period of assessment	Data in mean ± SD	Follow-up visit comparison	p-value (two-way ANOVA)
V0	10.86±19.74	V0-V1	0.014*
V1	7.64±17.52	V1-V2	0.055
V2	5.615±14.07	V2-V3	0.100
V3	4.036±10.64	V3-V0	0.001*
		V0-V2	0.001*
		V1-V3	0.001*

A split-plot repeated-measures ANOVA revealed no statistically significant interaction between sex and ulcer-healing pattern (F = 0.099, p = 0.757), indicating that TCC was equally effective in both males and females. Total contact casting showed significant improvement in the healing of plantar ulcers in both the forefoot and hindfoot.

## Discussion

Plantar foot ulcers are a common complication of peripheral neuropathy. Offloading is the technique used to treat plantar ulcers. It can be achieved through complete bed rest, different crutches, a walker, a cane, healing sandals, or TCC, with or without a walking iron.

Most patients who attended our OPD meet the criteria for TCC. We conducted an observational study from March 2024 to August 2024. In this study, we investigated the effectiveness of TCC in treating plantar ulcers. We included 16 patients aged 40 to 60 years, of whom 12 had forefoot ulcers, and four had hindfoot plantar ulcers.

Lazzarini et al. demonstrated that non-removable knee-high offloading devices such as the TCC are the first choice for healing forefoot and midfoot neuropathic plantar ulcers [12]. Begg et al. showed a significant reduction in plantar contact area via TCC compared with the shoe cast. With TCC, there is a significant reduction in plantar peak pressure beneath the midfoot and forefoot [13].

We performed TCC for plantar ulcers and observed its effectiveness in treating them. Our study showed a significant reduction in plantar ulcer volumes from the first visit to the subsequent visit (baseline vs. first follow-up after two weeks of TCC: p-value = 0.003, baseline vs. second follow-up at six weeks after TCC: p-value = 0.033).

Sahu et al. reported that ulcers treated with TCC took longer to heal (a mean of 48 ± 7 days) by six to seven casts, whereas ulcers treated with traditional dressings took an average of 58 ± 9 days to heal. The high efficacy of the TCC, along with its low risk of significant complications, makes TCC a gold-standard procedure for treating neuropathic foot ulcers [14]. Messenger et al. showed that the standard off-loading therapy in appropriate diabetic foot ulcer (DFU) management aligns with evidence-based guidance [15].

Wukich and Motko reported that 14 minor complications occurred among 82 consecutive patients who received TCC (closed-toe), but no significant complications occurred. Most of the complications were skin-related pressure ulcers, and another complication was that one patient perceived the cast as tight, so there was an earlier visit and a cast change. No iatrogenic skin problem was caused by the cast being too tight [16]. In our study, we observed that TCC significantly improved the healing of plantar ulcers, with a low complication rate.

Saha et al., in their study, showed that 22 patients of 56 (39.29%) in group A (occlusive dressings and TCC) had complete healing of the ulcer, as well as 11 patients of 52 (21.15%) in group B (only TCC). Subgroup analysis of complete responders showed that the outcome of the treatment was not influenced by age, sex, or rural/urban status. [17]. We too did not find any significant gender predominance affecting ulcer healing.

Li et al. found in their study that TCC in DFU patients improved ulcer-healing rates and shortened healing time compared with removable walking casts and footwear, but also reported an increased prevalence of complications [18]. We encountered no significant complications in our study. Our study found that TCC plays an effective role in the healing of plantar ulcers.

In their study, Zhang et al. found that most patients (84.9%) had complete healing or were healing their ulcers, with an average healing time of 81.5 days. Despite this efficacy, a large proportion of patients with recurrent ulcers (47.4%) remained, which aligns with the literature, which indicates a 65% recurrence rate at three to five years [19]. In our study, two patients reported ulcer recurrence, which was treated with TCC again. The factors that precipitated the ulcers to develop in the first place (poor wound care, neuropathy, and ischemia) continue to lead to further ulcers, and vigilant monitoring is needed.

Our study showed that TCC may help in the healing of hindfoot ulcers. To the best of our knowledge, we did not find any literature showing the role of TCC on hindfoot ulcers. Thus, we hope to add the literature regarding the same. The limitation of our study is that it has a small sample size, as we did not find many patients with hindfoot ulcers, and we did not compare healing between the groups. (forefoot vs. hindfoot). Also, there was no control group. Furthermore, our study is single-centered, with limited long-term follow-up.

## Conclusions

Total contact casting may be an effective, non-invasive method for healing chronic non-healing plantar ulcers, both forefoot and hindfoot, though it is time-consuming and associated with minimal complications. It effectively reduced plantar pressure, as considerable weight is borne by the TCC walls. It requires good patient compliance, as multiple visits are required.
